# Primary hyperoxaluria and systemic oxalosis

**DOI:** 10.4103/0970-1591.30276

**Published:** 2007

**Authors:** K. Sriram, Nitin S. Kekre, Ganesh Gopalakrishnan

**Affiliations:** Department of Urology, Christian Medical College, Vellore, Tamilnadu, India

Primary hyperoxaluria (PH) is an autosomal recessive disorder of glyoxylate metabolism, characterized by an excessive production and urinary excretion of oxalate and glyoxylate (PH Type1 or PH1) or oxalate and L-glycerate (PH Type2 or PH2).

The basic defect in PH1 is a functional defect of hepatic enzyme, alanine: glyoxylate amino transferase (AGT). On the other hand, PH2 is characterized by a deficiency of glyoxylate reductase/hydroxypyruvate reductase (GRHPR).[1]

**Figure 1:**
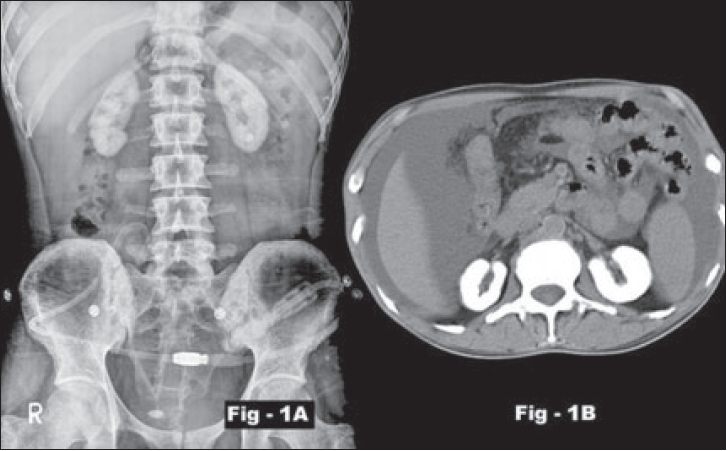
Plain X-ray KUB region (Fig. 1 A) and noncontrast CT abdomen (Fig. 1B) showing calcified kidneys

The basic problems in primary hyperoxaluria are caused by low solubility of Calcium oxalate (CaOx), which is deposited in the kidney and the urinary tract as nephrocalcinosis or urolithiasis [Figure 1A, B]. This leads to renal failure at which point, the increased production of oxalate is compounded by the failure to remove it from the body resulting in a very high corporeal oxalate load leading onto CaOx deposition all over the body. An international registry for hyperoxaluria is operational for these patients since 2004.[2]

**Figure 2:**
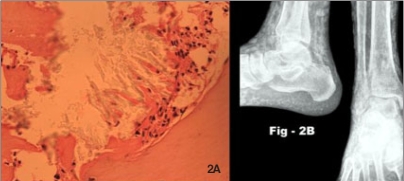
A. Bone marrow biopsy showing scanty marrow with collections of radially arranged refractile crystals suggestive of oxalate crystals with surrounding moderate fibrosis (40× magnification). B. Extensive oxalate crystal deposition in the soft tissues

Deposition of oxalate in various extra-renal tissues leading to a systemic involvement is named as systemic oxalosis.[3] Bone marrow is one of the major compartments of the insoluble oxalate pool [Figure 2A]. The other organs involved include soft tissue, heart, nerves, joints, skin, retina and other visceral lesions [Figure 2B].

The renal abnormalities include nephrocalcinosis caused by intratubular deposition of oxalate crystals. Plain radiographs may demonstrate an increasing density of the kidneys. On ultrasonography, the cortical echogenicity is increased early in the course of the disease, but with the onset of medullary calcification, the entire kidney becomes echogenic and the cortico-medullary differentiation is lost.

Computed tomography scan can detect the medullary and cortical calcifications even before they are evident on plain radiographs. Computed tomography attenuation values as high as 500 Houndsfield units have been reported. A global calcification of both the cortex and the medulla is characteristic of oxalosis. Mottled or speckled nephrocalcinosis may also be seen with methoxy flurane anesthesia and ethylene glycol poisoning. Diffuse cortical nephrocalcinosis is also seen in paraneoplastic hypercalciuria, Alport's syndrome, Acquired immunodeficiency syndrome-associated infections such as cytomegalovirus, mycobacterium avium-intracellulare infections. Medullary nephrocalcinosis is associated with medullary sponge kidney, hyperparathyroidism and renal tubular acidosis.

Soft tissue calcification can also occur in progressive systemic sclerosis, parasitic infestations, calcifying cavernous hemangioma, heterotopic calcifications after burn injuries, in tumor calcinosis and in the Thibierge-Weissenbach syndrome.[4] Each of these conditions is very rare and only a very few anecdotal reports are available in the literature. Crystalluria and infrared spectroscopy are useful in the identification and quantitative analysis of the crystals. However, a definitive diagnosis requires an assessment of enzyme assays in the liver tissue, facilities for which are not available in our country. The purpose of this Uro-radiology report is to emphasize that a combination of hyperoxaluria with nephrocalcinosis, extensive soft tissue calcification and progressive renal failure is sufficient to establish the diagnosis of systemic oxalosis obviating the need for enzyme assays in liver biopsy.
